# Negative association between dietary copper intake and human papillomavirus infection: A cross-sectional analysis of the National Health and Nutrition Examination Survey

**DOI:** 10.1371/journal.pone.0333901

**Published:** 2025-10-13

**Authors:** Chengya Feng, Xiaohe Lu, Zimian Fan, Xinxing Wang

**Affiliations:** 1 Department of Clinical Laboratory, Deyang Jingyang Maternal and Child Health Hospital, Deyang, Sichuan, China; 2 Department of Laboratory Medicine, Affiliated Hospital of Shandong University of Traditional Chinese Medicine, Jinan, Shandong, China; 3 Chengdu Sixth People’s Hospital, Chengdu, Sichuan, China; 4 Department of Clinical Laboratory, Chengdu BOE Hospital, Chengdu, Sichuan, China; UFRN: Universidade Federal do Rio Grande do Norte, BRAZIL

## Abstract

**Background:**

Human papillomavirus (HPV) is the most prevalent sexually transmitted infection. Copper is essential for immune function, but its association with HPV infection remains unclear. This study aims to investigate the relationship between dietary copper intake and HPV infection.

**Methods:**

This cross-sectional study analyzed 8,071 participants from the National Health and Nutrition Examination Survey (2003–2016). Copper intake was assessed using two 24-hour recalls, and HPV status was confirmed by DNA testing. Weighted multivariable logistic regression and restricted cubic splines (RCS) were used.

**Results:**

After adjusting for multiple confounders, dietary copper intake was significantly inversely associated with vaginal HPV infection (odds ratio [OR], 0.79; 95% confidence interval [CI], 0.67–0.92). Compared with women in the lowest quartile of dietary copper intake, those in the highest quartile had a lower adjusted OR for vaginal HPV infection (OR, 0.60; 95% CI, 0.48–0.73). RCS analysis revealed an L-shaped association with a threshold at 1.2 mg/day of copper intake. Subgroup analyses showed that marital status moderated the association between copper intake and HPV infection (*P* for interaction < 0.001), with significance in those married or living with a partner but not in those living alone.

**Conclusion:**

An L-shaped association was observed between copper intake and HPV infection, suggesting that maintaining an optimal level of copper intake may be associated with reduced risk of HPV infection and related diseases.

## Introduction

Human papillomavirus (HPV) has emerged as one of the most significant global health challenges, representing the most prevalent sexually transmitted infection with profound clinical consequences. As the etiological agent responsible for over 99% of cervical cancer cases, HPV contributes to more than 300,000 annual deaths worldwide, with particularly heavy disease burdens observed in low- and middle-income countries where screening infrastructure remains limited [1–3]. Beyond its well-established role in cervical carcinogenesis, HPV infection demonstrates concerning associations with various anogenital and oropharyngeal malignancies, while also causing substantial reproductive morbidity and psychosocial distress among affected individuals [4–6].

The development of prophylactic vaccines has undoubtedly transformed HPV prevention paradigms, yet significant limitations persist that necessitate complementary approaches. While vaccination programs in developed nations have achieved notable success in reducing vaccine-type HPV incidence [7,8], three critical gaps remain: First, economic and logistical barriers continue to limit vaccination coverage in many regions [9,10]. Second, the vaccines demonstrate no therapeutic efficacy against established infections [11,12]. Most importantly, approximately one-third of oncogenic HPV types fall outside current vaccine protection, sustaining persistent infection and subsequent malignancy risks [12].

These limitations have stimulated growing interest in alternative prevention strategies, particularly through modifiable lifestyle factors such as nutrition. Among potential dietary components, copper presents a particularly compelling yet understudied candidate due to its dual physiological roles [13]. As an essential trace element, copper serves as a critical cofactor for numerous enzymes involved in fundamental biological processes including energy metabolism, antioxidant defense, and neurotransmitter synthesis [14]. Its unique redox properties enable participation in both beneficial immune responses and potentially harmful oxidative pathways.

Copper is an essential nutrient for optimal immune function and has been shown to possess antiviral properties [15,16]. Studies have demonstrated that copper deficiency in rodents leads to decreased CD4 T-cell counts [16] and impairs specific CD4 T-cell responses to the HPV E6 protein, which may be critical for HPV clearance [17]. Moreover, copper metabolism within cells may directly interact with the HPV life cycle. For instance, copper ions can modulate the intracellular redox state, which may significantly affect HPV replication and transcription [18]. Excess or deficiency of copper ions may influence HPV infection efficiency and viral load by altering intracellular oxidative stress levels.

Notably, research specifically examining dietary copper intake in relation to HPV infection remains surprisingly limited, despite copper’s obligatory dependence on external sources since it cannot be endogenously synthesized or degraded [19]. Previous investigations have predominantly focused on serum copper levels or tissue concentrations, which may not accurately reflect long-term nutritional status. Furthermore, existing studies have generally failed to account for potential effect modification by behavioral factors such as sexual activity patterns, despite well-documented associations between sexual behavior and HPV acquisition risk.

To address these critical knowledge gaps, the primary aim of this study is to use data from the National Health and Nutrition Examination Survey (NHANES) to examine the relationship between dietary copper intake and HPV infection. Specifically, we aim to determine whether there is an optimal range of dietary copper intake for HPV prevention, while considering important potential confounders and effect modifiers.

## Methods

### Ethics statement

This study involved human participants and/or tissue and was conducted according to the guidelines laid down in the Declaration of Helsinki. The NHANES survey protocol was approved by the National Center for Health Statistics (NCHS) Research Ethics Review Board (ERB). All participants provided written informed consent.

### Type of study

This is a cross-sectional analysis of seven consecutive 2-year cycles of the NHANES conducted from 2003 through 2016 to examine the association between dietary copper intake and prevalent HPV infection among U.S. women.

### Study area

The data originate from the ongoing NHANES, a nationally representative program that employs a complex, stratified, multistage probability cluster sampling design in which primary sampling units are geographic counties (n = 30 per cycle) selected across the entire United States, followed by random selection of households and individuals within strata with deliberate oversampling of minority groups, the elderly, and low-income populations to enhance statistical power; all data collection activities—household interviews, Mobile Examination Center visits, and laboratory analyses—were integrated across the United States under the standardized NHANES protocol.

### Study design

A survey-weighted, complex sampling framework—incorporating stratification (SDMVSTRA), clustering (SDMVPSU), and the dietary two-day integrated weight (WTDR2D) to account for differential selection probabilities, non-response bias in dietary recalls, and post-stratification to U.S. Census benchmarks—was applied to yield nationally representative estimates.

### Study sample

**Fig 1:**
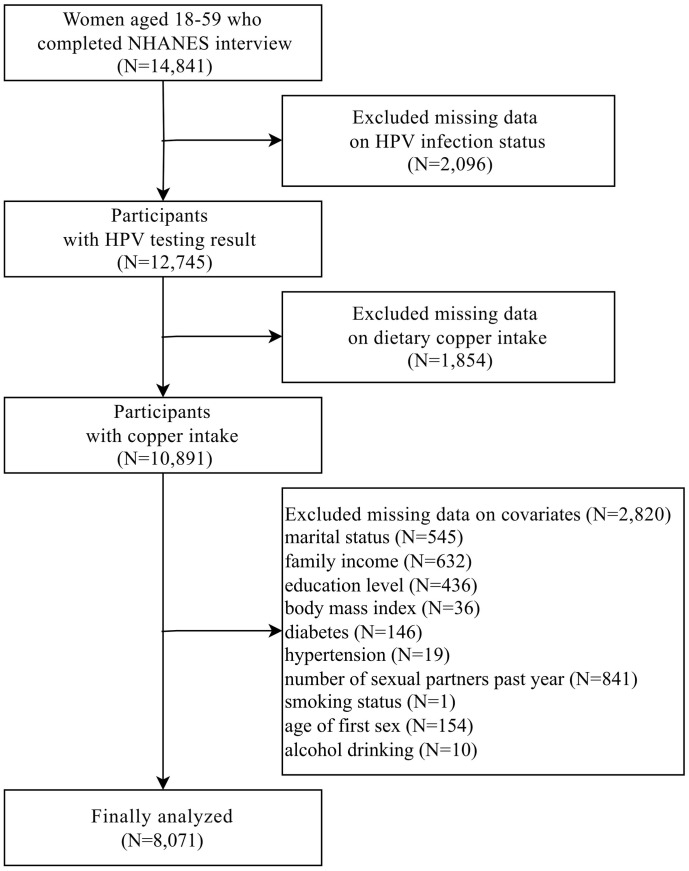
Flow diagram of the screening and enrollment of study participants. NHANES, National Health and Nutrition Examination Survey; HPV, human papillomavirus.

Female participants aged 18–59 years across the 2003–2016 NHANES cycles who had information on genital HPV infection constituted the source population (n = 14,841); participants were sequentially excluded if they lacked data on genital HPV infection status (n = 2,096), dietary copper intake (n = 1,854), or any of the required covariates—marital status (n = 545), family income (n = 632), education level (n = 436), body mass index (n = 36), diabetes (n = 146), hypertension (n = 19), number of sexual partners in the past year (n = 841), smoking status (n = 1), age of first sex (n = 154), and alcohol drinking (n = 10)—yielding a final analytic sample of 8,071 women with a mean age of 40.0 (SE 0.3) years and a mean age at first sexual intercourse was 18.2 (SE 0.4) years, participant flow is depicted in Fig 1.

### Principles of the study

The study adhered to the NHANES analytical guidelines and adopted a complete-case approach for the primary analysis. The anonymity of the publicly released data was maintained throughout.

### Data provenance and sources

Anonymized data were downloaded directly from the NHANES website, and survey weights were applied to account for nonresponse and ensure national representativeness; no additional primary data collection was performed.

### Exposure assessment – dietary copper intake

Data on dietary copper intake were derived from two nonconsecutive 24-hour dietary recalls in the NHANES. The first recall was conducted in a mobile examination center, and the second was administered via telephone 3 to 10 days later. Both recalls were facilitated by a computer-assisted dietary interview system. Copper concentrations in foods (unit: mg/100g) were obtained from the corresponding versions of the United States Department of Agriculture (USDA)‘s Food and Nutrition Database for Dietary Studies (version 1.0 for 2003–2004, up to version 7.0 for 2015–2016), which directly integrated nutrient data from the USDA Standard Reference legacy database. The total daily copper intake was determined by averaging the copper intake values from the two separate 24-hour dietary recall surveys, excluding dietary supplements. Composite foods were broken down using USDA recipe files. Dietary copper intake was stratified into quartiles: Q1 ranged from 0 to 0.76 mg/day, Q2 from 0.77 to 1.02 mg/day, Q3 from 1.03 to 1.34 mg/day, and Q4 from 1.35 mg/day and above.

### Outcome

In this study, we utilized the Roche Linear Array HPV Genotyping Test to detect 37 types of HPV from self-collected vaginal swab samples. Participants collected samples using standard vaginal swabs, which were then placed in SurePath preservative fluid and transported on dry ice to the CDC laboratory at −70°C. DNA was extracted using the QIAamp DNA Mini Kit (concentration ≥50 ng/μL). HPV L1 gene and β-globin internal control were amplified by PCR using PGMY09/11 primers. The amplified products were subjected to reverse line blot hybridization using the BeeBlot automated system to detect 37 types of HPV (6, 11, 16, 18, 26, 31, 33, 35, 39, 40, 42, 45, 51, 52, 53, 54, 55, 56, 58, 59, 61, 62, 64, 66, 67, 68, 69, 70, 71, 72, 73, 81, 82, 83, 84, 89, IS39). Quality control measures included β-globin positivity verification (>5 copies, samples not meeting the criteria were excluded), retesting of HPV52-positive samples using the LightCycler 480 quantitative PCR (threshold of ≥5 copies/10 μL to exclude cross-reactivity with HPV33/35/58). According to NHANES protocol, each strip was read independently by two technologists blinded to each other; discrepancies were resolved by a senior reviewer, with inclusion of negative and positive controls in each batch (containing 50 copies of HPV16 plasmid). A positive result for any HPV type was defined as a positive HPV infection (binary variable). For detailed methodology, refer to the Roche manual and the NHANES laboratory handbook.

### Covariates

Based on previous scientific literature and clinical insights, we evaluated a range of potential covariates, including age, race, education level, marital status, family income, body mass index (BMI), diabetes, hypertension, alcohol drinking, smoking status, age of first sex, and number of sexual partners in the past year. Race was categorized into non-Hispanic White, non-Hispanic Black, Mexican American, or other races. Education level was divided into three groups based on years of education: less than 9 years, 9–12 years, or more than 12 years. Marital status was classified as living alone (never married, separated, divorced, or widowed) or living with a partner (married or cohabiting). Family income was stratified into three levels according to the poverty-income ratio (PIR): low-income (PIR ≤ 1.3), middle-income (PIR = 1.3–3.5), and high-income (PIR > 3.5). BMI (kg/m²) was calculated using standardized anthropometric measurements collected during the health examination, defined as weight (kg) divided by height (m) squared. The presence of preexisting conditions, such as hypertension and diabetes, was determined based on self-reported diagnoses by physicians or health professionals. Alcohol drinking status was assessed by whether participants consumed at least 12 drinks of any type of alcoholic beverage in any given year. Smoking status was classified into three categories: never smokers (fewer than 100 cigarettes), current smokers, and former smokers (those who quit smoking after consuming more than 100 cigarettes). Sexual history was ascertained from responses to the sexual behavior questionnaire, with the number of sexual partners in the past year categorized as 0, 1, or 2 or more. Additionally, we assessed collinearity among the covariates by calculating the variance inflation factor and examining the correlation matrix. All VIF values were below 10, and none of the correlation coefficients reached statistical significance. This indicates that there is no substantial collinearity among the selected covariates, ensuring the stability of the model estimates and the reliability of the results.

### Statistical analyses

Consistent with NHANES analytical guidelines, we used the previously described complex survey features—stratification (SDMVSTRA), clustering (SDMVPSU), and the WTDR2D weight—to obtain nationally representative estimates. Our primary analysis employed a complete-case approach after excluding participants with missing data on any covariate. Categorical variables are presented as unweighted counts (weighted percentages), and continuous variables are presented as means ± standard error. Group differences for continuous variables were assessed using one-way ANOVA, and associations for categorical variables were assessed using chi-square tests. Weighted multivariable logistic regression was used to calculate the odds ratios (OR) and 95% confidence intervals (CI) for the association between dietary copper intake and vaginal HPV infection. We defined four models: Model 1 was unadjusted; Model 2 adjusted for age, race, education level, marital status, and family income; Model 3 added BMI, diabetes, hypertension, alcohol use, and smoking status to Model 2; and Model 4 further incorporated age of first sex and number of sexual partners in the past year into Model 3. To explore potential effect modification, we generated forest plots for interactions across different subgroups. A Bonferroni correction was applied to account for multiple comparisons in the five prespecified subgroup analyses (age, marital status, family income, alcohol drinking, and number of sexual partners). Linearity assumptions for continuous variables were assessed using restricted cubic splines (RCS) with knots at the 10th, 50th, and 90th percentiles of dietary copper intake, optimized via Akaike Information Criterion minimization. Influential observations were evaluated through residual-leverage contour plots incorporating predefined Cook’s distance thresholds (critical d = 0.5) (S1 Fig). Given documented limitations of the Hosmer–Lemeshow test in large samples [20], model fit was assessed using LOESS calibration curves comparing predicted probabilities against observed event rates (S2 Fig).

Robustness was evaluated by three sensitivity analyses: exclusion of participants with cancer to remove immune compromise, exclusion of vaccinated participants to eliminate vaccine-induced bias, and multiple imputation (5 replications, chained equations via R mice procedure) to address missing-data bias; no substantive changes in conclusions were observed.

All statistical analyses were conducted using R Statistical Software (Version 4.2.2, http://www.R-project.org, The R Foundation) and Free Statistics Analysis Platform (Version 2.1.1, Beijing, China, http://www.clinicalscientists.cn/free statistics).

## Results

We employed several analytical strategies to examine the relationship between copper intake and HPV infection. First, we used multivariable regression models to assess the overall association. Second, we used nonlinear models to identify potential threshold effects. Finally, we conducted sensitivity analyses to test the robustness of our findings.

### Characteristics of the participants

The baseline characteristics of excluded participants (those with missing covariate data) and included participants (those with complete covariate data) are presented in S1 Table. Overall, the baseline data appear stable; however, we cannot rule out the potential impact of non-random missing data on the results. Table 1 summarizes the baseline characteristics of the 8,071 participants, stratified by quartiles of dietary copper intake. Participants in higher intake quartiles were generally older, more likely to be Non-Hispanic White, married or living with a partner, and had higher educational attainment and family income. Health outcomes and behaviors also varied significantly across quartiles. Notably, mean BMI and the proportion of current smokers were highest in the lowest quartile (Q1: 29.3 kg/m² and 34.8%, respectively) and lowest in the highest quartile (Q4: 28.1 kg/m² and 15.1%, respectively) (*P* < 0.001). The prevalence of hypertension was also lowest in Q4 (14.1%, *P* = 0.008). No significant difference was observed for diabetes across the groups (*P* = 0.140).

**Table 1:** Baseline characteristics of participants stratified by dietary copper intake quartiles.

Variables	Dietary copper intake (mg/day)					
	Total	Q1(≤0.76)	Q2(0.77–1.02)	Q3(1.03–1.34)	Q4(≥1.35)	*P* value
No.	8,071	1,874	2,014	2,067	2,116	
**Demographic**						
Age (years)	40.0 (0.3)	38.8 (0.4)	39.7 (0.4)	40.6 (0.4)	40.7 (0.4)	0.004
Race/ethnicity						<0.001
Non-Hispanic White	3,573 (67.6)	761 (61.7)	849 (65.5)	932 (69.1)	1,031 (72.4)	
Non-Hispanic Black	1,770 (12.3)	552 (18.9)	470 (13.1)	401 (10.6)	347 (8.3)	
Mexican American	1,321 (8.3)	253 (7.7)	358 (9.1)	375 (9.3)	335 (7.2)	
Others	1,407 (11.8)	308 (11.8)	337 (12.3)	359 (11.1)	403 (12.0)	
Education level (years)						<0.001
< 9	434 (3.1)	127 (4.7)	120 (3.7)	117 (3.0)	70 (1.5)	
9 - 12	2,702 (29.9)	827 (41.6)	754 (34.6)	620 (27.1)	501 (19.7)	
> 12	4,935 (67.0)	920 (53.8)	1,140 (61.6)	1,330 (69.9)	1,545 (78.8)	
Marital status						<0.001
Married or living with a partner	4,919 (65.0)	989 (56.7)	1,232 (64.9)	1,303 (67.9)	1,395 (68.9)	
Living alone	3,152 (35.0)	885 (43.3)	782 (35.2)	764 (32.1)	721 (31.1)	
Family income						<0.001
Low	2,568 (22.5)	811 (33.9)	663 (24.7)	582 (19.5)	512 (14.7)	
Medium	2,808 (32.9)	671 (35.6)	722 (33.4)	722 (31.8)	693 (31.6)	
High	2,695 (44.6)	392 (30.5)	629 (41.9)	763 (48.6)	911 (53.7)	
**Health Status**						
Diabetes	567 (6.0)	151 (6.9)	144 (6.0)	153 (6.9)	119 (4.7)	0.140
Hypertension	1,470 (17.3)	395 (18.5)	382 (18.0)	376 (19.3)	317 (14.1)	0.008
Vaginal HPV	3,545 (40.5)	1,005 (52.7)	922 (43.6)	829 (35.3)	789 (33.3)	<0.001
BMI (kg/m^2^)	28.9 (0.2)	29.3 (0.3)	29.1 (0.2)	29.2 (0.3)	28.1 (0.2)	<0.001
Alcohol drinking	5,433 (73.4)	1,231 (69.9)	1,365 (74.2)	1,381 (71.9)	1,456 (76.8)	0.003
Smoking status						<0.001
Never	5,048 (59.8)	998 (50.7)	1,239 (57.6)	1,399 (64.6)	1,412 (64.2)	
Former	1,303 (18.0)	264 (14.5)	329 (17.6)	322 (18.3)	388 (20.7)	
Current	1,720 (22.2)	612 (34.8)	446 (24.8)	346 (17.1)	316 (15.1)	
**Sexual activity**						
Age of first sex	18.2 (0.4)	16.9 (0.1)	17.4 (0.1)	19.2 (0.8)	18.9 (1.0)	<0.001
Number of sexual partners in the past year						0.002
0	1,209 (14.2)	289 (15.0)	307 (13.8)	308 (14.2)	305 (14.1)	
1	5,815 (73.8)	1,286 (69.0)	1,457 (74.4)	1,514 (76.0)	1,558 (74.8)	
≥ 2	1,047 (12.0)	299 (16.0)	250 (11.9)	245 (9.8)	253 (11.2)	

BMI, body mass index; Q1Q4, quartiles based on dietary copper consumption; HPV, human papillomavirus.

### Association between dietary copper intake and HPV infection

Univariate analysis showed that marital status, family income, alcohol consumption, age at first sex, and the number of sexual partners in the past year were associated with HPV infection (S2 Table).

Table 2 presents the results of multivariate regression analyses examining the association between dietary copper intake and vaginal HPV infection. When dietary copper was modeled as a continuous variable, each 1 mg/day increase in intake was associated with a significantly reduced odds of HPV infection after full adjustment for demographic, socioeconomic, health behavior, and sexual activity covariates (OR = 0.79; 95% CI: 0.67–0.92; *P* = 0.003). When analyzed by quartiles, a significant inverse dose-response trend was observed across increasing quartiles of copper intake (*P* for trend <0.001). Compared to the lowest quartile (Q1, ≤ 0.76 mg/day), the fully adjusted ORs for Q2, Q3, and Q4 were 0.83 (95% CI: 0.67–1.03; *P* = 0.087), 0.65 (95% CI: 0.53–0.81; *P* < 0.001), and 0.60 (95% CI: 0.49–0.75; *P* < 0.001), respectively. The strength of the association was attenuated but remained significant with sequential adjustment for confounders, most notably in Q4.

**Table 2:** Dietary copper intake associated with vaginal HPV infection status.

	Model 1		Model 2		Model 3		Model 4	
	OR (95% CI)	*P* value	OR (95% CI)	*P* value	OR (95% CI)	*P* value	OR (95% CI)	*P* value
Copper intake (mg/day)	0.65 (0.56-0.76)	<0.001	0.76 (0.65-0.89)	<0.001	0.79 (0.67-0.92)	0.003	0.79 (0.67-0.92)	0.003
Copper intake quartile								
Q1(≤0.76)	Reference		Reference		Reference		Reference	
Q2 (0.77–1.02)	0.70 (0.57-0.84)	<0.001	0.80 (0.65-0.98)	0.03	0.81 (0.66-1.00)	0.052	0.83 (0.67-1.03)	0.087
Q3 (1.03–1.34)	0.49 (0.40-0.60)	<0.001	0.60 (0.48-0.74)	<0.001	0.64 (0.51-0.78)	<0.001	0.65 (0.53-0.81)	<0.001
Q4 (≥1.35)	0.45 (0.37-0.54)	<0.001	0.57 (0.46-0.70)	<0.001	0.60 (0.48-0.73)	<0.001	0.60 (0.49-0.75)	<0.001
p for trend		<0.001		<0.001		<0.001		<0.001

HPV, human papillomavirus. OR, odds ratio. CI, confidence interval. Q, quartile.

Model 1 not adjusted.

Model 2 adjusted for age, race, education level, marital status, family income.

Model 3 adjusted for model 2, additionally adjusted for body mass index, diabetes, hypertension, alcohol drinking, smoke status.

Model 4 adjusted for model 3, additionally adjusted for age of first sex, number of sexual partners in the past year.

**Fig 2:**
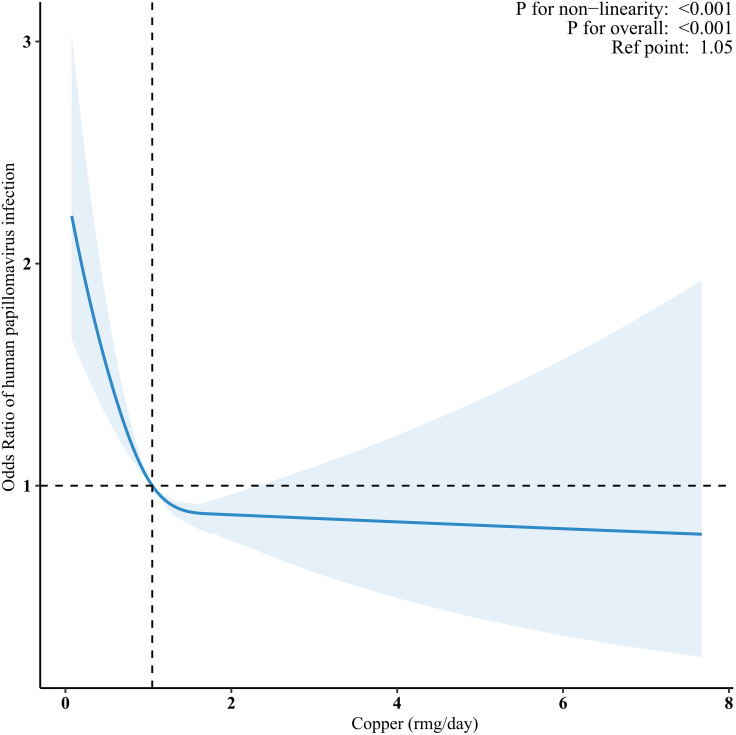
The dose-response relationship between dietary copper intake and the risk of HPV infection status. HPV, human papillomavirus. NHANES, National Health and Nutrition Examination. The blue solid line indicates the smooth curve fit between the variables, the shaded area surrounding the line represents the 95% confidence interval. The restricted cubic spline model was adjusted for age, race, education level, marital status, family income, body mass index, diabetes, hypertension, alcohol drinking, smoke status, age of first sex, number of sexual partners in the past year. The plot displays only 99% of the data.

RCS analysis demonstrated an L-shaped relationship between dietary copper intake and HPV infection (nonlinear, *P* < 0.001; Fig 2). Table 3 presents the results of the threshold analysis using a two-piecewise regression model, this analysis identified a nonlinear relationship between dietary copper intake and the likelihood of HPV infection. Participants with dietary copper intake ≤1.2 mg/day had 55% lower odds of HPV infection (adjusted OR 0.45; 95% CI 0.29–0.70; *P* < 0.001). However, when daily copper intake was equal to or greater than 1.2 mg/day, further increases in copper intake did not show a significant correlation with HPV infection prevalence (*P* = 0.743). This pattern suggests a saturable protective effect, where achieving a minimum intake level is critical, but exceeding it does not further reduce risk.

**Table 3:** Association between dietary copper intake and vaginal HPV infection status using two-piecewise regression models.

	Crude model		Adjusted model^*^	
Dietary copper intake (mg/day)	OR (95% CI)	*P* value	OR (95% CI)	*P* value
≤1.2	0.27 (0.18-0.41)	<0.001	0.45 (0.29-0.70)	<0.001
>1.2	1.00 (0.88-1.13)	0.972	0.97 (0.83-1.14)	0.743

HPV, human papillomavirus. OR, odds ratio. CI, confidence interval.

* Adjusted for age, race, education level, marital status, family income, body mass index, diabetes, hypertension, alcohol drinking, smoke status, age of first sex, number of sexual partners in the past year.

### Subgroup analysis and sensitivity analyses

**Fig 3:**
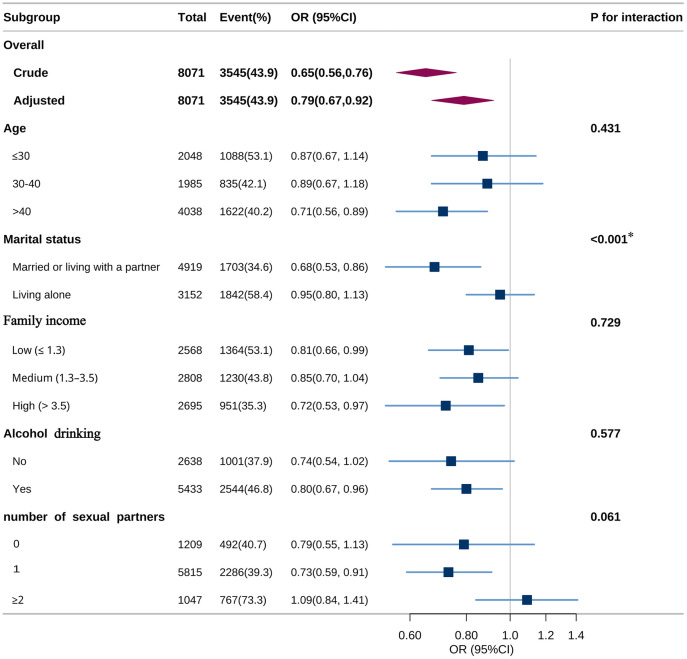
Association between dietary copper intake and vaginal HPV infection status according to the general characteristics. HPV, human papillomavirus. OR, odds ratio. CI, confidence interval. * *P* value for interaction is significant after Bonferroni correction. Except for the stratification factor itself, the stratifications were adjusted for all variables (age, race, education level, marital status, family income, body mass index, diabetes, hypertension, alcohol drinking, smoke status, age of first sex, number of sexual partners in the past year).

A stratified analysis assessed potential effect modification of dietary copper intake on HPV infection across multiple subgroups. After stratifying by age, marital status, family income, alcohol drinking, and number of sexual partners in the past year, after Bonferroni correction for five subgroup comparisons (α = 0.01), only marital status demonstrated a statistically significant interaction effect (adjusted *P* = 0.005). This modification effect was observed in individuals who were married or living with a partner (OR: 0.68, 95% CI: 0.53–0.86) but not in those living alone (OR: 0.95, 95% CI: 0.80–1.13). No significant interactions were observed in other subgroups after multiplicity adjustment (Fig 3).

Table 4 summarizes the results of sensitivity analyses performed to assess the robustness of the association between dietary copper intake and HPV infection. After full adjustment, the association remained significant across all scenarios: after excluding participants with cancer (adjusted OR = 0.79; 95% CI: 0.68–0.93), after excluding HPV-vaccinated individuals (adjusted OR = 0.75; 95% CI: 0.60–0.93), and after multiple imputation for missing data (adjusted OR = 0.85; 95% CI: 0.76–0.98). These results demonstrate the consistency of the protective association under different analytical assumptions.

**Table 4:** Sensitivity analyses.

Analysis	Sample size	Crude model		Adjusted model	
		OR (95% CI)	*P* value	OR (95% CI)	*P* value
Excluding cancer participants	7611	0.67 (0.57 - 0.78)	<0.001	0.79 (0.68 - 0.93)	0.004
Excluding HPV-vaccinated participants	5266	0.58(0.46 - 0.73)	<0.001	0.75(0.60 - 0.93)	0.01
Multiple imputation for missing covariates	10891	0.70(0.60 - 0.83)	<0.001	0.85(0.73 - 0.98)	0.028

HPV, human papillomavirus. OR, odds ratio. CI, confidence interval.

Crude model: adjusted for no covariates.

Adjusted model: adjusted for age, race, education level, marital status, family income, body mass index, diabetes, hypertension, alcohol drinking, smoke status, age of first sex, number of sexual partners in the past year.

## Discussion

The association between dietary micronutrients and HPV infection, a primary cause of cervical cancer, remains an area of active investigation. This study aimed to explore whether dietary copper intake is associated with the prevalence of vaginal HPV infection among U.S. women. In this nationally representative large cross-sectional study, after adjusting for potential confounders, an inverse correlation was found between dietary copper intake and vaginal HPV infection in American women, with a threshold saturation effect at 1.2 mg/day. This association remained significant after excluding cancer patients and those vaccinated against HPV, as well as after applying multiple imputation for missing covariate data. Subgroup analysis revealed a stronger link among individuals who are married or living with a partner.

While the immunomodulatory functions of copper are widely recognized, epidemiological research on the association between dietary copper and HPV infection remains limited. Two key studies provide preliminary evidence: Barchitta et al., based on a cross-sectional study in Italy, found that Mediterranean dietary patterns rich in copper (such as nuts and legumes) were significantly associated with a reduced risk of persistent high-risk HPV infection [21]. Xiao et al. demonstrated that adequate dietary copper intake was associated with a lower prevalence of high-risk HPV infection, although the dose-response relationship was not explored [22]. Building on this foundation, the present study quantified this non-linear relationship using RCS modeling. Furthermore, subgroup analyses revealed that the protective effect of copper was more pronounced among women who were married or living with a partner. These findings provide novel epidemiological evidence for developing targeted dietary copper intervention strategies to prevent HPV infection.

Copper plays a complex role in viral infections, and its association mechanisms with HPV have not yet been fully elucidated, potentially involving multiple metabolic pathways. Existing research has demonstrated that viral infections can disrupt host copper metabolism, induce the production of reactive oxygen species, and subsequently trigger oxidative stress and autophagy [23,24]. Moreover, viral infections can also interfere with the host cell’s endocytic pathways, such as the sorting mechanisms mediated by SNX27, leading to abnormal localization and metabolic disturbances of copper transporters ATP7A (ATPase, Cu^2+^ transporting, alpha polypeptide) and ATP7B (ATPase, Cu++ transporting, beta polypeptide) [25]. Additionally, copper ions may exert direct antimicrobial effects by disrupting the protein structures of pathogens [26]. Copper transporters ATP7A/B, which are transported by the molecular chaperone protein Atox1, are core molecules in regulating copper homeostasis [27]. Maintaining copper homeostasis is crucial for supporting antiviral immunity, and systemic copper imbalances may impact immune function. Given the importance of copper in immunity, maintaining copper homeostasis may help reduce the risk of persistent HPV infection. We posit that the interplay between viral infections and copper metabolism will provide novel insights into HPV-related clinical syndromes and pave the way for antiviral strategies targeting the copper pathway. Furthermore, adequate copper intake is associated with reduced HPV prevalence, though causality requires verification via intervention studies.

The study has several strengths. Utilizing the weighted NHANES database ensures that the results are representative of women in the United States, providing objective evidence for the prevention and treatment of HPV infections. The research highlights that individuals who are married or living with a partner may have a greater need for copper intake, offering a basis for targeted interventions in high-risk HPV populations. Additionally, the study identifies a threshold for copper intake, which can accurately guide daily copper consumption. Overall, these findings lay a solid foundation for future research on the relationship between dietary copper and HPV infection.

This study has several limitations. First, the cross-sectional NHANES design inherently precludes causal inference between copper intake and HPV infection. Critically, we cannot establish temporal precedence—specifically, whether low copper levels causally preceded HPV exposure or were a consequence of metabolic alterations related to infection. Reverse causation remains plausible, as HPV-induced physiological changes may alter dietary behaviors and nutrient absorption. Second, because of the single-time HPV status measurement, we cannot assess the impact of copper intake on HPV acquisition or persistence; large-scale prospective studies are needed. Third, despite comprehensive adjustment for covariates, residual confounding may persist from unmeasured factors, including partners’ HPV status and precise zinc intake, given the biological interplay between zinc and copper. Fourth, while NHANES sampling weights ensure nationally representative estimates for non-institutionalized U.S. adult women, these findings are not generalizable to other populations—including men, non-U.S. cohorts, or clinical subgroups—without external validation. Fifth, inter-rater reliability (κ) for HPV genotyping could not be determined because the dual-reader worksheets were not retained. To address these constraints, future research should: (1) implement prospective cohorts establishing temporal relationships while controlling reverse causation; (2) conduct RCTs testing copper supplementation in identified high-risk subgroups (e.g., married/cohabiting women); and (3) conduct external validation in men, non-U.S. populations, and diverse clinical subgroups to establish the generalizability of the copper–HPV association.

## Conclusions

The findings of this cross-sectional study indicate that dietary copper intake is negatively correlated with HPV infection in American women. Individuals who are married or living with a partner may have a greater need for copper intake. These results may provide a novel basis and direction for future research investigating factors associated with reduced risk of HPV infections.

## Supporting information

S1 Fig Residual-leverage plot with Cook’s distance contours for influential point assessment. This plot illustrates the relationship between leverage values and standardized residuals. The green dashed lines represent Cook’s distance contours, with all data points falling within these contours – thus ruling out influential observations.
(TIF)

S2 Fig LOESS calibration curve for the final multivariable model. The smoothed LOESS line closely tracks the ideal calibration line, indicating excellent agreement between predicted probabilities and observed event rates. Calibration slope = 1.00 (95% CI 0.94–1.06), intercept = 0.00 (95% CI –0.05–0.05).
(TIF)

S1 Table The basic characteristics of the excluded (n = 2820) and included (n = 8071) participants. BMI, body mass index. Q1–Q4, quartiles based on dietary copper consumption. HPV, human papillomavirus.
(DOCX)

S2 Table Association of covariates and HPV status in women. HPV, human papillomavirus. OR, Odds Ratio. CI, Confidence Interval.
(DOCX)

## Abbreviations

HPV: human papillomavirus
NHANES: National Health and Nutrition Examination Survey
RCS: restricted cubic splines
DNA: deoxyribonucleic acid
BMI: body mass index
PIR: poverty-income ratio
OR: odds ratios
CI: confidence interval
USDA: United States Department of Agriculture
ATP7A: ATPase, Cu^2+^ transporting, alpha polypeptide
ATP7B: ATPase, Cu^2+^transporting, beta polypeptide
