# Supplementary figures and images for: Negative association between dietary copper intake and human papillomavirus infection: A cross-sectional analysis of the National Health and Nutrition Examination Survey
Source: PLoS One. 2025 Oct 13;20(10):e0333901. doi: 10.1371/journal.pone.0333901 (PMC12517482; doi:10.1371/journal.pone.0333901)

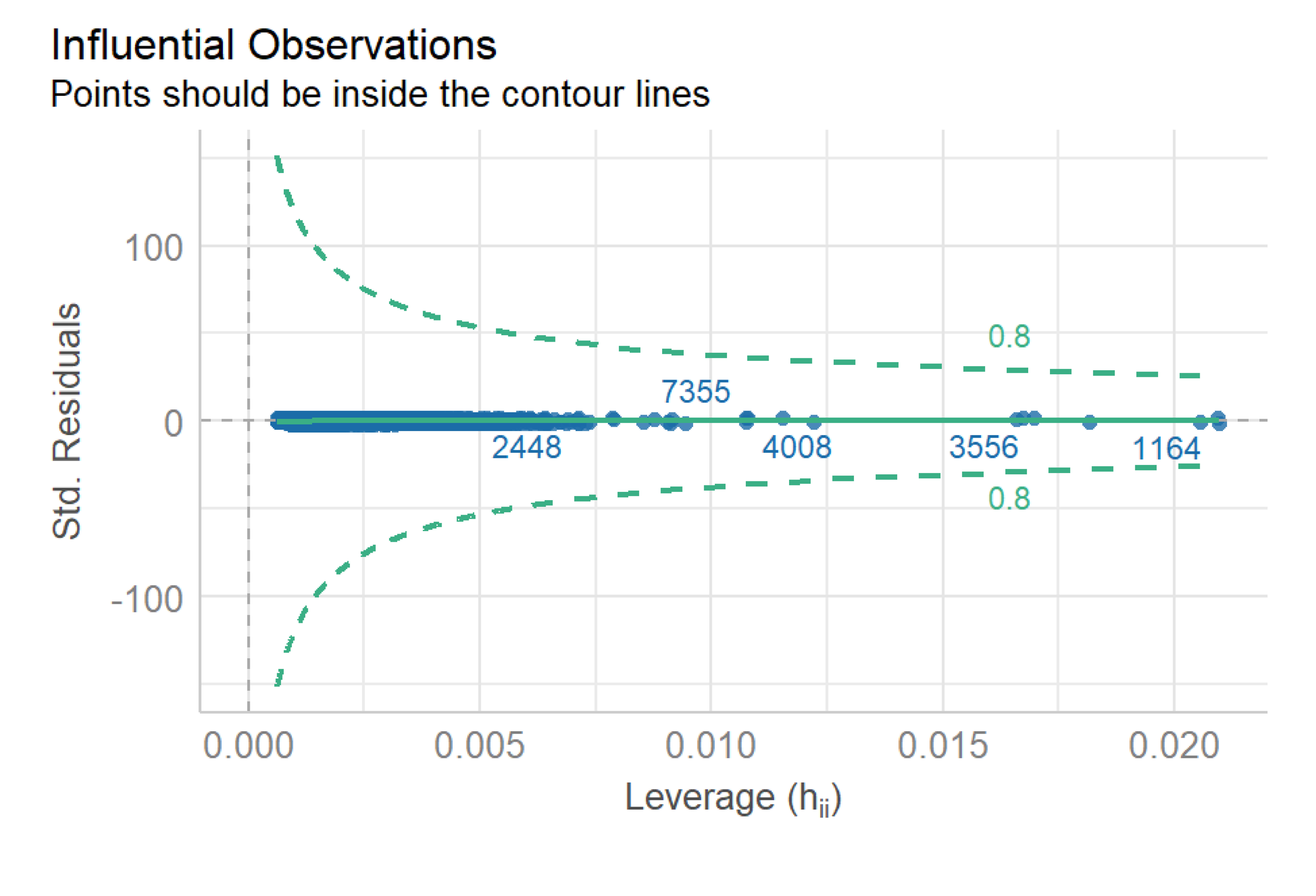

Supplement: S1 Fig — This plot illustrates the relationship between leverage values and standardized residuals. The green dashed lines represent Cook’s distance contours, with all data points falling within these contours – thus ruling out influential observations. (TIF) [file pone.0333901.s001.tif]

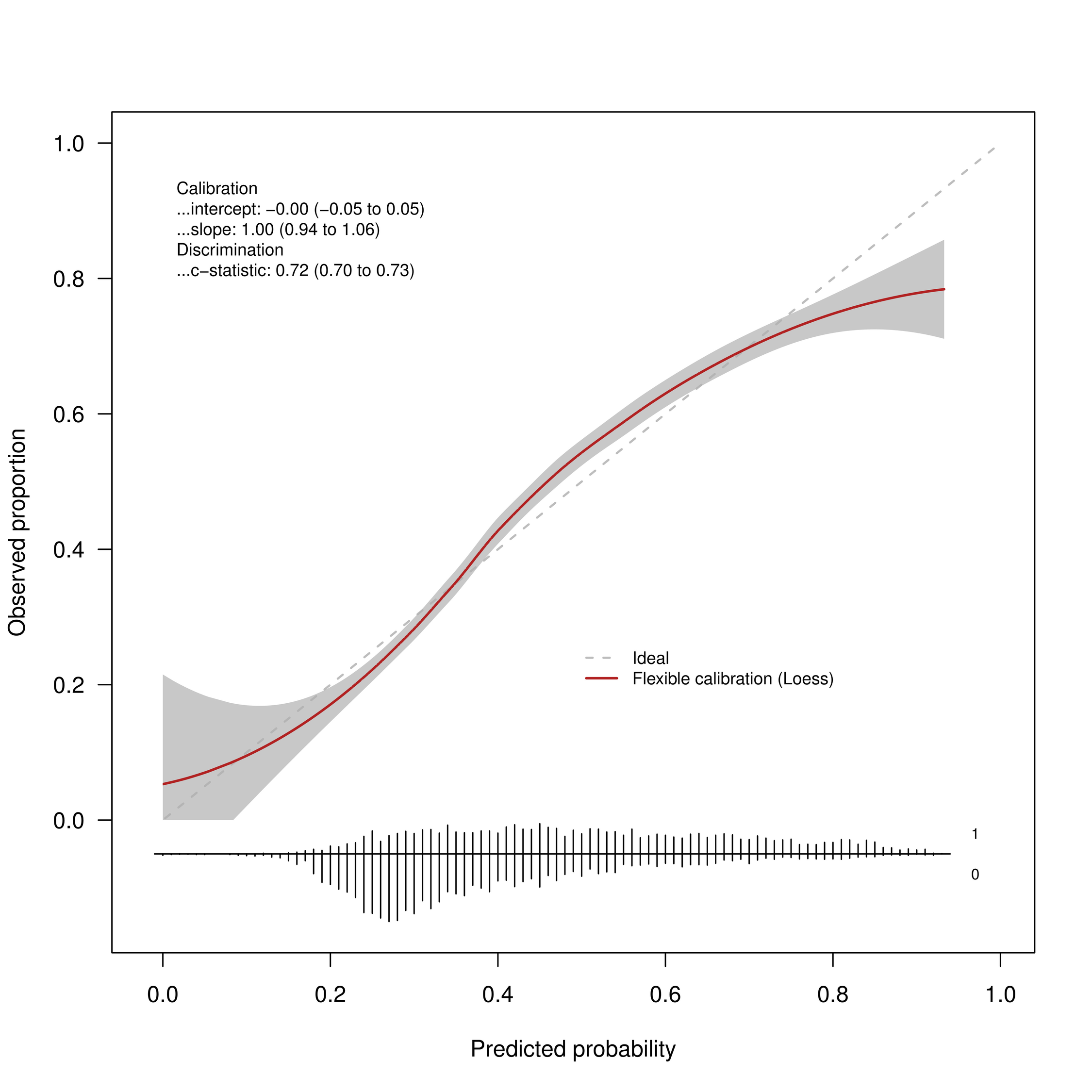

Supplement: S2 Fig — The smoothed LOESS line closely tracks the ideal calibration line, indicating excellent agreement between predicted probabilities and observed event rates. Calibration slope = 1.00 (95% CI 0.94–1.06), intercept = 0.00 (95% CI –0.05–0.05). (TIF) [file pone.0333901.s002.tif]
