# Supplementary material for: Negative association between dietary copper intake and human papillomavirus infection: A cross-sectional analysis of the National Health and Nutrition Examination Survey
Source: PLoS One. 2025 Oct 13;20(10):e0333901. doi: 10.1371/journal.pone.0333901 (PMC12517482; doi:10.1371/journal.pone.0333901)
Supplement: S1 Table — BMI, body mass index. Q1–Q4, quartiles based on dietary copper consumption. HPV, human papillomavirus. (DOCX) [file pone.0333901.s003.docx]

**S1 Table** The basic characteristics of the excluded (n = 2820) and included (n = 8071) participants.

| Variables | Total  (n = 10891) | Covariates Missing  (n = 2820) | Covariates Complete  (n = 8071) | *P* value |
| --- | --- | --- | --- | --- |
| **Demographic** |  |  |  |  |
| Age (years) | 37.3 ± 12.4 | 32.0 ± 13.6 | 39.2 ± 11.3 | < 0.001 |
| Race/ethnicity |  |  |  | < 0.001 |
| Non-Hispanic White | 4348 (39.9) | 775 (27.5) | 3573 (44.3) |  |
| Non-­Hispanic Black | 2501 (23.0) | 731 (25.9) | 1770 (21.9) |  |
| Mexican American | 2029 (18.6) | 708 (25.1) | 1321 (16.4) |  |
| Others | 2013 (18.5) | 606 (21.5) | 1407 (17.4) |  |
| Education level (years) | |  |  | < 0.001 |
| <9 | 703 ( 7.1) | 269 (14.8) | 434 (5.4) |  |
| 9 - 12 | 3378 (34.2) | 676 (37.3) | 2702 (33.5) |  |
| >12 | 5802 (58.7) | 867 (47.8) | 4935 (61.1) |  |
| Marital status | |  |  | < 0.001 |
| Married or living with a partner | 5986 (57.9) | 1067 (46.9) | 4919 (60.9) |  |
| Living alone | 4360 (42.1) | 1208 (53.1) | 3152 (39.1) |  |
| Family income^c^ |  |  |  | < 0.001 |
| Low | 3619 (35.5) | 1051 (49.2) | 2568 (31.8) |  |
| Medium | 3491 (34.2) | 683 (32) | 2808 (34.8) |  |
| High | 3098 (30.3) | 403 (18.9) | 2695 (33.4) |  |
| **Health Status** |  |  |  |  |
| Vaginal HPV | 4791 (44.0) | 1246 (44.2) | 3545 (43.9) | 0.81 |
| Diabetes | 685 ( 6.4) | 118 (4.4) | 567 (7) | < 0.001 |
| Hypertension | 1836 (16.9) | 366 (13.1) | 1470 (18.2) | < 0.001 |
| Alcohol drinking | | |  | < 0.001 |
| No | 3404 (35.4) | 766 (50) | 2638 (32.7) |  |
| Yes | 6200 (64.6) | 767 (50) | 5433 (67.3) |  |
| Smoking status | |  |  | < 0.001 |
| Never | 6523 (64.5) | 1475 (72.1) | 5048 (62.5) |  |
| Former | 1523 (15.1) | 220 (10.8) | 1303 (16.1) |  |
| Current | 2070 (20.5) | 350 (17.1) | 1720 (21.3) |  |
| BMI (kg/m^2^) | 29.3 ± 7.8 | 28.5 ± 7.7 | 29.6 ± 7.8 | < 0.001 |
| Copper (mg/day) | 1.1 ± 0.7 | 1.1 ± 0.6 | 1.1 ± 0.7 | < 0.001 |
| **Sexual activity** |  |  |  |  |
| Age of first sex | 17.0 (15.0, 19.0) | 17.0 (15.0, 18.0) | 17.0 (15.0, 19.0) | < 0.001 |
| Number of sexual partners past year | | |  | < 0.001 |
| 0 | 1639 (17.5) | 430 (32.7) | 1209 (15) |  |
| 1 | 6518 (69.5) | 703 (53.5) | 5815 (72) |  |
| ≥2 | 1228 (13.1) | 181 (13.8) | 1047 (13) |  |

BMI, body mass index. Q1–­Q4, quartiles based on dietary copper consumption. HPV, human papillomavirus.
