# Supplementary material for: Negative association between dietary copper intake and human papillomavirus infection: A cross-sectional analysis of the National Health and Nutrition Examination Survey
Source: PLoS One. 2025 Oct 13;20(10):e0333901. doi: 10.1371/journal.pone.0333901 (PMC12517482; doi:10.1371/journal.pone.0333901)
Supplement: S2 Table — HPV, human papillomavirus. OR, Odds Ratio. CI, Confidence Interval. (DOCX) [file pone.0333901.s004.docx]

**S2 Table** Association of covariates and HPV status in women

| Variable | OR (95%CI) | *P* value |
| --- | --- | --- |
| Age(years) | 0.98 (0.97-0.98) | <0.001 |
| Race/ethnicity |  |  |
| Non-Hispanic White | 1 (Reference) |  |
| Non-­Hispanic Black | 2.62 (2.28-3.02) | <0.001 |
| Mexican American | 1.03 (0.82-1.30) | 0.798 |
| Others | 1.06 (0.88-1.27) | 0.532 |
| Education level (years) |  |  |
| <9 | 1 (Reference) |  |
| 9 - 12 | 1.29 (0.93-1.79) | 0.131 |
| >12 | 0.9 (0.63-1.28) | 0.552 |
| Marital status |  |  |
| Married or living with a partner | 1 (Reference) |  |
| Living alone | 2.83 (2.51-3.20) | <0.001 |
| Family income |  |  |
| Low (≤ 1.3) | 1 (Reference) |  |
| Medium(1.3­3.5) | 0.61 (0.53-0.71) | <0.001 |
| High (> 3.5) | 0.42 (0.36-0.50) | <0.001 |
| Diabetes |  |  |
| No | 1 (Reference) |  |
| Yes | 0.75 (0.58-0.97) | 0.028 |
| Hypertension |  |  |
| No | 1 (Reference) |  |
| Yes | 0.95 (0.78-1.17) | 0.629 |
| Body mass index (kg/m^2^) | 1.00 (0.99-1.01) | 0.885 |
| Alcohol drinking |  |  |
| No | 1 (Reference) |  |
| Yes | 1.41 (1.21-1.64) | <0.001 |
| Smoking status |  |  |
| Never | 1 (Reference) |  |
| Former | 1.02 (0.85-1.23) | 0.82 |
| Current | 2.25 (1.90-2.66) | <0.001 |
| Age of first sex | 0.91 (0.89-0.93) | <0.001 |
| Number of sexual partners past year |  |  |
| 0 | 1 (Reference) |  |
| 1 | 0.95 (0.79-1.15) | 0.601 |
| ≥2 | 4.09 (3.06-5.46) | <0.001 |

HPV, human papillomavirus. OR, Odds Ratio. CI, Confidence Interval.
